# We Should Improve Personalization of Management in Patients with a Diagnosis of Schizophrenia

**DOI:** 10.3390/jcm11010184

**Published:** 2021-12-30

**Authors:** Alfonso Tortorella

**Affiliations:** Department of Psychiatry, University of Perugia, 06132 Perugia, Italy; alfonso.tortorella@unipg.it

**Keywords:** schizophrenia, diagnosis, negative symptoms, cognitive function, social skills training, physical comorbidities, childhood abuse, internalized stigma

## Abstract

The current management of patients with schizophrenia is marked by a lack of personalization. After the diagnosis is made, a second-generation antipsychotic is usually prescribed based on the current clinician’s preferences, sometimes accompanied by a psychosocial intervention which is typically not evidence-based and not targeted to the specific needs of the individual patient. In this opinion paper, some steps are outlined that could be taken in order to address this lack of personalization. A special emphasis is laid on the clinical characterization of the patient who has received a diagnosis of schizophrenia. Considerations are put forward concerning the assessment of the negative dimension in ordinary clinical practice, which is often neglected; the evaluation of cognitive functioning using a simple test battery which requires limited professional training and takes no more than 15 min to administer; the evaluation of social functioning using a validated instrument focusing on personal care skills, interpersonal relationships, social acceptability, activities, and work skills; and the assessment of the unmet needs of the person (including practical, social, and emotional needs, and existential or personal recovery). The implications of the assessment of these domains for the formulation of the management plan are discussed.

The opinion I put forward in this paper is that the current management of patients with schizophrenia is marked, in several clinical contexts worldwide, by a considerable lack of personalization, and that much can be done to address this situation.

After a diagnosis of schizophrenia is made, often without referring to formal diagnostic systems [1,2], the management is often stereotyped, with the prescription of a second-generation antipsychotic based on the current preferences of the clinician [3] and sometimes the addition of a psychosocial intervention which may not be evidence-based and not targeted to the specific needs of the individual patient [4]. A psychotherapeutic intervention is rarely considered, despite currently available evidence [5,6,7]. Here I will briefly outline some steps that could be taken in order to address this lack of personalization. The paper is intended for clinicians worldwide, although it is understood that there are several contexts in which significant advances have been already made in the personalization of management of patients with schizophrenia, e.g., [8], and others in which the available resources will allow the implementation of only part of the steps indicated.

The first level to be considered is that of diagnosis. The term “schizophrenia” is often misused to refer to any primary psychosis (i.e., any psychosis which is not due to the effects of a substance or a medication and not secondary to another medical condition or mood disorder) or even to any psychosis. Conversely, there are clinical contexts in which the term “schizophrenia” is avoided, mostly due to the stigmatizing connotation that it has assumed [9], and the generic term “psychosis” is used as a synonym for “schizophrenia”. These practices are of course incorrect and are currently obscuring the clinically crucial problem of differential diagnosis with respect to psychoses. The diagnosis of schizophrenia should be based on the current conceptualization of the syndrome, as it emerges from official diagnostic systems.

The second level to be considered is that of clinical characterization. The diagnosis of schizophrenia, as any other diagnosis in psychiatry, is not sufficient in itself to guide the formulation of the management plan [10]. It has to be complemented by a more detailed clinical characterization of the individual case on the basis of a series of domains that have been recently listed and described by a group of experts [11].

The first domain is that of psychopathological dimensions [12,13,14]. The negative dimension is particularly neglected in ordinary clinical practice, although some of its elements (in particular, poor emotional expression and avolition) have been reported to be strong predictors of several outcome measures, including socialization, participation in family life, behaviour in emergency situations, social contacts, and need for treatment [15].

Clinicians should become familiar with the actual contents of this dimension: affective blunting (i.e., a reduction in the expression of emotion and in reactivity to events); alogia (i.e., a reduction in the quantity of spoken words and the amount of information spontaneously given when answering a question); asociality (i.e., a reduction in social interactions and initiative), anhedonia (i.e., a reduction either in the experience or in the anticipation of pleasure), and avolition (i.e., a poor engagement in any activity due to lack of interest and motivation) [16,17,18,19,20].

There are now several rating scales for negative symptoms. One could argue that almost all of them are too detailed, take too much time to administer, and require too much training such that they are not suitable for use in ordinary clinical practice. However, there is at least one exception: the Brief Negative Symptom Scale (BNSS) [21], which is a very simple validated rating scale consisting of just 13 items which can be used in ordinary practice without much training and takes about 20 min to administer.

After the negative dimension has been characterized in the individual patient, it is first of all important to clarify whether negative symptoms are secondary or primary. In fact, in many cases, negative symptoms are secondary to other illness dimensions, such as positive symptoms, depression, extrapyramidal symptoms, sedation, environmental deprivation, or substance use. So, these elements should be considered in the individual patient, and if one of them emerges prominently as a likely explanation for the negative symptoms, then we should address this element in the management plan and it can be expected that negative symptoms will consequently improve.

We have today several non-pharmacological interventions validated for use in negative symptoms, including social skills training, cognitive behavioural therapy (CBT), and cognitive training, although their impact on primary negative symptoms remains to be tested in controlled trials [22,23,24,25]. These non-pharmacological interventions are implemented in several contexts worldwide, including rehabilitation day centers.

On the pharmacological side, there is just one antipsychotic which has been proved to be superior to another antipsychotic in treating primary negative symptoms. This is cariprazine in comparison with risperidone; however, there is just one study of it and no independent replication is available [26,27,28]. There are several studies concerning the impact of various antipsychotics on negative symptoms but they do not concern specifically primary negative symptoms.

A second domain which should be considered in the patient with schizophrenia is that of cognitive impairment [29,30,31,32,33]. In fact, according to currently available evidence, neurocognition is the strongest predictor of real-life social functioning in the future in psychotic patients. In the follow-up phase of the multicenter study of the Italian Network for Research on Psychoses, neurocognition at baseline was the most powerful predictor of everyday life skills at follow-up, a significant predictor of work skills at follow-up, and—mostly through social cognition—a strong predictor of interpersonal relationships at follow-up [34].

The neurocognitive processes that are most likely to be impaired in patients with schizophrenia are: speed of processing (i.e., the speed with which simple perceptual and motor tasks can be performed); verbal learning and memory (i.e., encoding, recognition, and recall of information involving language); visuospatial learning and memory (i.e., encoding, recognition, and recall of visuospatial information); working memory (i.e., temporary maintenance and manipulation of information in consciousness); attention/vigilance (i.e., ability to sustain a focus on relevant information over a prolonged period of time); reasoning and problem solving (i.e., strategic and logical thinking, planning, formation and maintenance of goals, and the coordination of these processes flexibly over time) [29,35,36,37,38].

The assessment of neurocognition in patients with schizophrenia in ordinary clinical practice remains today an open issue. In the Diagnostic and Statistical Manual of Mental Disorders (DSM-5), if we look at the chapter on psychotic disorders, it seems that the assessment of neurocognitive processes can simply be a part of the clinical interview, whereas if we look at Section 3 of the manual it seems that the use of a neuropsychological test battery is advised but no specific neuropsychological test battery is mentioned.

Indeed, there are many neuropsychological test batteries validated for use in psychotic patients [39,40,41,42,43,44,45]. One could argue that most of them require too much professional training and take too much time to administer, thus not being suitable for use in ordinary clinical practice.

However, there are now some tools which require limited professional training, usually available online, and which take 10–15 min to administer. Two of them are interview-based, so they are closer to the style to which the clinician is accustomed. They are the Brief Cognitive Assessment Tool for Schizophrenia [46], which takes about 10 min to administer; the Cognitive Assessment Interview [47], which is interview-based and takes about 15 min to administer; and the Schizophrenia Cognition Rating Scale [48], which is interview-based and takes about 15 min to administer.

We have now two validated interventions targeting neurocognitive impairment in schizophrenia. They are cognitive remediation, aerobic exercise, and their combination. There are several approaches to cognitive remediation, whose core features include using cognitive training techniques, usually computerized; therapist-guided refinement of problem-solving strategies; and facilitation of the transfer of cognitive strategies to daily life. Effect sizes have been reliably demonstrated to be medium for cognitive improvements [49]. It is very important to emphasize that both these interventions can be personalized, i.e., they can be tailored to the needs of the individual patient. They should be personalized on the basis of the profile of neurocognitive impairment emerging from the characterization that we have just mentioned [36,49,50,51,52,53,54,55,56,57].

I will now focus on two further related areas, that of social functioning and that of the patient’s unmet needs. According once again to the results of the multicenter study of the Italian Network for Research on Psychoses [34], everyday life skills and functional capacity are at the core of the schizophrenia network, being the two nodes that are most central and most interconnected, whereas, for instance, positive symptoms represent a node which is more remote and less interconnected. Furthermore, according to the only study available using a machine learning approach to predict both short-term and medium-term treatment outcomes in patients with first-episode psychosis, the strongest predictors of both end points were all psychosocial in nature, including unemployment, poor education, functional deficits, and unmet psychosocial needs [58].

Social functioning can be assessed in patients with schizophrenia in ordinary clinical practice using a very well validated instrument, the Specific Level of Functioning Scale (SLOF), which takes just 30–40 min to administer and can be used without very extensive training. This tool is very simple, with five main subscales focusing on personal care skills, interpersonal relationships, social acceptability, activities, and work skills [59].

We have several validated social skills training interventions available for patients with schizophrenia but they are often used in a way that is stereotyped. The same protocol is applied to all patients, whereas the intervention can be personalized, it can be tailored on the basis of the profile of social dysfunction emerging from the abovementioned characterization of the individual patient [60].

Furthermore, if from that characterization it emerges that a lack of motivation is a prominent aspect in that individual patient, then you cannot expect social skills training to be effective [61]. In these cases, my advice is to use PRIME, a mobile app intervention validated for use in order to improve motivation, and only when the lack of motivation is at least in part corrected is social skills training to be applied [62].

We will focus now on the related area of patient’s unmet needs. Every clinician will acknowledge that patients’ unmet needs, in particular the unmet needs of psychotic patients, are important. However, this aspect is not commonly addressed systematically in ordinary clinical practice in order to guide the formulation of the management plan. The unmet needs of persons with schizophrenia can be actually subdivided into two categories: practical, social, and emotional needs; and the so-called existential or personal recovery [63,64].

The first category includes unmet needs, such as housing, food, cleaning, self-care, daytime activities, information on illness and treatment, social relationships, sexual life, education, security, financial tasks, employment, and social benefits. The expression existential recovery encompasses such aspects as the restoration of the sense of oneself or one’s identity, of one’s autonomy, of a perspective for the future, the feeling that life is meaningful and worth living [65].

For the systematic assessment of patients’ practical, emotional and social needs, we have an instrument which has been translated into many languages and used for many years, which is the Camberwell Assessment of Need (CAN) [66,67,68], whereas for the evaluation of existential or personal recovery my advice is to use the Recovery Assessment Scale [69]. The systematic characterization of the practical, emotional, and social needs of the individual patient with a diagnosis of schizophrenia will have important implications for the formulation of the management plan, of course in collaboration with the patient. For instance, if unemployment emerges as a prominent unmet need, then the Individual Placement and Support (IPS) model is an intervention which has been validated in many countries and cultural contexts [70,71,72].

It is more complex to address the area of personal or existential recovery. There is the need for a more in-depth and intense shared decision making process with the patient, and, in addition to this, there is often the need to reconsider and readjust the characteristics of the therapeutic environment. In fact, while most clinicians will probably argue that their mental health service is recovery-oriented, this is not what emerges from the evaluation by patients themselves in ordinary clinical practice [65].

We will consider now an area whose importance most clinicians will acknowledge but which is often not concretely taken into account in the clinical characterization of the patient with a diagnosis of schizophrenia and in the formulation of the relevant management plan in ordinary clinical practice. This is the domain of physical comorbidities. All clinicians are now aware that patients with schizophrenia are at increased risk for many physical diseases, particularly prominent among them being cardiovascular diseases and diabetes mellitus, and many clinicians will at least have heard of one of the sets of guidelines produced by various organizations and associations during the past ten to fifteen years concerning the examinations to be done at baseline and then at different points of time during the follow-up of patients with schizophrenia [73,74,75,76,77,78,79,80]. However, the fact is that, unfortunately, in ordinary clinical practice, these guidelines are not frequently implemented.

Furthermore, while most clinicians will acknowledge that second-generation antipsychotics are not at all interchangeable with each other concerning their impact on physical health [27,81,82], it is not common in ordinary clinical practice for the choice of antipsychotic to be made on the basis of these considerations. The antipsychotic is often chosen solely on the basis of the doctor’s preference at that particular point in time. Equally, the individual lifestyle counselling and psychoeducation interventions which should ideally be considered in all patients with schizophrenia in order to promote a healthier lifestyle, and which should certainly be considered if risk factors or actual manifestations of physical diseases emerge from the clinical characterization, are not commonly available and used in ordinary clinical practice. We argue that they should be available and used in all mental health services [83,84].

I will now consider briefly a domain very rarely considered in the clinical characterization of patients with schizophrenia which is aimed at the formulation of a personalized management plan. This is the domain of early environmental exposures.

Probably not many clinicians are aware that one of the three or four strongest non-genetic risk factors for primary psychosis is a history of childhood maltreatment and that this history is a powerful predictor of a poor response to treatment, so that it may represent an undetected source of what is called treatment resistance [85,86,87,88].

We have a very simple instrument available, the Childhood Trauma Questionnaire (CTQ) [89], whose administration takes just 10–15 min, and which can be used in ordinary clinical practice in order to assess reliably and reasonably this patient aspect. In fact, if a history of childhood trauma is prominent in the case of a particular patient, then our management will have to be particularly intensive and careful because there will be a higher risk of non-adherence and consequently non-response to both pharmacological and non-pharmacological interventions. In some of these patients, one of the validated trauma-focused CBT-based psychological interventions may be indicated [90].

I will now finally consider a domain that is acknowledged by all clinicians but which is very rarely considered in the context of the clinical characterization of the individual patient with schizophrenia aimed at the personalization of the management plan. This is the domain of internalized stigma.

It is well known that patients with schizophrenia tend to internalize social stigma and discrimination. Probably less known is that this internalized stigma may have a powerful negative impact on help-seeking and on adherence and consequently response to pharmacological and non-pharmacological interventions [91,92,93,94].

Today this aspect can be assessed reliably and reasonably in ordinary clinical practice using a validated instrument called the Internalized Stigma of Mental Illness Scale [95,96]. If this aspect emerges prominently, we could consider one of the validated group interventions, mostly with a psychoeducational component, targeting this aspect. Moreover, we will have to consider and possibly adjust the family environment and, in some cases, also the therapeutic environment, because internalized stigma may be in part iatrogenic, so that some aspects of the therapeutic relationships in that particular service may need to be reconsidered [97].

In conclusion, the management of patients with primary psychosis is today in several contexts remarkably stereotyped. What is usually done is to make a diagnosis of psychosis or schizophrenia and just on that basis to indiscriminately prescribe a second-generation antipsychotic, sometimes accompanied by a psychosocial intervention which is often non-systematic, non-personalized, and non-evidence based. This practice should be overhauled. The management of schizophrenia should become less stereotyped and more personalized. Diagnosis should always be complemented by a more detailed clinical characterization of the individual patient, covering at least the domains that I have briefly considered here.
